# Number of intra-operative cyclic knee motion required to achieve stable graft tension in anterior cruciate ligament reconstruction; a prospective clinical study

**DOI:** 10.1186/s12891-024-07239-x

**Published:** 2024-02-05

**Authors:** Manato Horii, Ryuichiro Akagi, Shotaro Watanabe, Takahiro Enomoto, Hiroaki Hosokawa, Seiji Ohtori, Takahisa Sasho

**Affiliations:** 1 Department of Orthopedic Surgery, Graduate School of Medicine, Chiba University, 1-8-1 Inohana, Chuo-ku, Chiba, Chiba 260-8670 Japan; 2 Oyumino Central Hospital, Knee Surgery and Sports Medicine Center, 6-49-9 Oyumino-Minami, Midori-Ku, Chiba, Chiba 266-0033 Japan; 3 Department of Orthopedics, Toho University Sakura Medical Center, 564-1 Shimoshizu, Sakura, Chiba, 285-8741 Japan; 4 Center for Preventive Medicine, Musculoskeletal Disease and Pain, Chiba University, 1-8-1 Inohana, Chuo-ku, Chiba, Chiba 260-8670 Japan

**Keywords:** Anterior cruciate ligament reconstruction, Cyclic knee motion, Graft tension, Hamstrings

## Abstract

**Background:**

Applying pretension by cyclic knee motion immediately before graft fixation in anterior cruciate ligament (ACL) reconstruction surgery decreases graft elongation during the postoperative course. However, the expected change in graft tension caused by cyclic knee motion remains unclear. We measured graft tension changes caused by cyclic knee motion during double-bundle ACL reconstruction.

**Methods:**

We included 39 patients undergoing primary anatomical double-bundle ACL reconstruction with autologous hamstrings as graft sources, at multiple centers between February 2021 and August 2022. After securing the anteromedial (AM) and posterolateral (PL) bundle grafts to the femoral cortex, they were initially tensioned to 40 N per bundle. After 10 cycles of knee extension and flexion motion, ranging from 0 to 90–110°, tension was re-measured and re-tensioned to 40 N if the graft tension had decreased. This was repeated thrice for 10 cycles on each graft. Every 10 cycles, we recorded graft tension changes (ΔGT) and compared the mean ΔGT in the AM and PL bundles. Furthermore, we assessed relationships between total ΔGT in each bundle, age, sex, and graft diameter.

**Results:**

Twenty-five women and 14 men with a mean age of 27.4 ± 12.4 years were included. The mean ΔGT in AM and PL bundles after every 10 cycles were 6.6 ± 3.7 N, 3.0 ± 2.3 N, 1.4 ± 1.5 N, and 9.9 ± 3.8 N, 4.9 ± 2.6 N, and 2.5 ± 1.9 N, respectively. There were significant differences in ΔGT in both bundles after every 10 cycles (*p* < 0.01). ΔGT in the AM bundle was significantly lower than in the PM bundle at the same number of cycles (*p* < 0.01). No correlation was observed between ΔGT in either bundle and age, sex, or graft diameter.

**Conclusions:**

The initially applied graft tension decreased by intra-operative cyclic knee movement, and the changes in graft tension decreased after retention and repeated cycles. Three sets of 10 cycles knee motion may avoid initial tension loss of the hamstring autograft in the early phase after double-bundle ACL reconstruction.

## Introduction

Adequate initial graft tension is an important factor affecting the clinical outcomes of anterior cruciate ligament (ACL) reconstruction [1]. Recent studies have reported that a larger graft tension may lead to over-constraining of the knee joint, resulting in a loss of range of motion and worse survival with the graft [2]. However, the results of another study suggested that lower graft tension may lead to post-operative knee laxity [3]. Creep of the graft caused by sustained tensile loading may cause a reduction in the initial graft tension and knee instability after ACL reconstruction [4].

The pretension technique is considered useful for properly adjusting the initial graft tension during ACL reconstruction [4, 5]. The cyclic or static load on the graft before fixation theoretically eliminates the loosening of the knot between the thread and the graft and stiffens the graft itself [6]. Consequently, the initial graft tension remains stable after ACL reconstruction. Previous reports have described the methodology and effectiveness of the pretension technique [5, 7]. A biomechanical study using a commercial tensiometer revealed that intra-articular pretension significantly minimized graft elongation before final graft fixation compared with the maximal manual pull of the graft [7]. Intra-operative repetition of flexion and extension of the knee joint is simple yet effective for pretension [2]. A previous study revealed that a four-strand hamstring tendon graft elongates after cyclic knee motion [5]. Despite reports demonstrating the relationship between cyclic knee motion and graft elongation, few have measured the changes in initial graft tension caused by cyclic knee motion. Applying pretension by cyclic knee motion immediately before graft fixation in ACL reconstruction surgery decreases graft elongation during the postoperative course. However, the expected change in graft tension caused by cyclic knee motion remains unclear.

This novel study measured changes in initial graft tension caused by cyclic knee motion in double-bundle ACL reconstruction. In addition, we elucidated the patient factors related to larger graft tension changes and hypothesized that graft tension would reduce by immediate cyclic knee motion.

## Methods

### Study design

This study was approved by the Certified Clinical Research Board of our hospital (M10609). Informed consent was obtained from all patients and guardians of minor patients. We conducted a prospective clinical study of ACL reconstruction at multiple centers between February 2021 and August 2022. Forty-three patients underwent primary anatomical double-bundle ACL reconstruction using a semitendinosus tendon autograft. The exclusion criteria were as follows: 1) concomitant knee fracture and other ligament injuries, 2) history of knee surgery or knee trauma, and 3) insufficient records. Patient characteristics such as age, sex, body mass index (BMI), and graft diameter were collected from the electric medical records.

### Surgical technique and measurement

Two orthopedic surgeons (R.A. and T.S.) with experience in knee surgery and three orthopedic residents (M.H., S.W., and H.H.) supervised by R.A. or T.S. performed the ACL reconstruction.

After conventional arthroscopic examination through the anteromedial (AM) and anterolateral portals, the hamstring autografts were harvested. An oblique incision of 3 cm in length was created on the anteromedial tibial surface at the pes anserinus level, and the semitendinosus tendon was harvested using an open-loop tendon stripper (Smith & Nephew Endoscopy, Andover, MA, USA). Subsequently, the harvested tendon was cut into halves and folded, creating two double-stranded bundles 6.0 cm or more in length, looped over the TightRope RT (Arthlex Inc., Naples, Florida). The open free ends of the graft were armed with a 1.3 mm Taperoop (Arthlex Inc., Naples, Florida). Traction was applied on the graft to tighten the suture during the graft preparation process. After preparation, the grafts were kept without further pretension until implantation.

﻿The position of the femoral tunnel was arthroscopically determined with reference to the intercondylar ridge, posterior femoral condyle cartilage edge, and femoral footprint of the remnant ACL [8]. The femoral AM tunnels were placed 6 mm anterior to the posterior femoral condyle cartilage edge and the femoral posterolateral (PL) tunnels were placed distal to the lateral bifurcate ridge and posterior to the intercondylar ridge using the outside-in technique. Additionally, the tibial bone tunnel site was arthroscopically determined with reference to the ACL remnant, medial tibial eminence, anterior horn of the lateral meniscus, and transverse ligament [9]. Tibial tunnels were positioned at the center of the AM and PL footprints. Both AM and PL grafts were introduced through each bone tunnel, and the TightRope RT button (Arthlex Inc., Naples, Florida, USA) was flipped outside the femoral cortex for fixation [10].

**Fig. 1:**
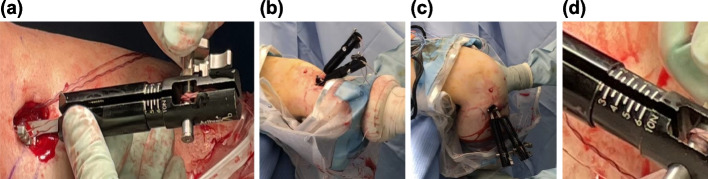
Measurement of graft tension change caused by cyclic knee motion during ACL reconstruction (**a**) the graft is fixed at an initial tension of 40 N using a tensiometer (Arthlex Inc., Naples, Florida) (**b**), (**c**) repeated knee flexion and extension between 0 degree and 90–110 degrees for 10 cycles (**d**) the graft tension is measured using the tensiometer

Once the femoral end of the graft was fixed, a 6.5 mm collar of the TensionLoc (Arthlex Inc., Naples, Florida) system was placed on the tibial cortex at each graft tunnel end, and the graft tension was measured using a tensiometer (Arthlex Inc., Naples, Florida, USA) before final fixation. An initial tension of 40 N was applied to the AM and PL bundles at a knee flexion of 30 ° and full knee extension, respectively. Subsequently, the knee joint was moved from full extension to 90 °–110 °flexion for 10 cycles [5] with the tension meter applying tension on the graft during the procedure﻿ (Fig. 1). The tension value indicated by the tension meter was recorded after 10 cycles of knee motion. If a decrease in graft tension was observed, the graft was re-tensioned to 40 N. This set of tension measurements and the knee cyclic motion procedure was repeated until a total of 30 cycles were applied to each graft, and the graft tension was recorded every 10 cycles. Finally, each graft was re-tensioned to 40 N and fixed using the TensionLoc plug (Arthlex Inc., Naples, Florida, USA). The femoral cortical fixations were confirmed to have no gap between the femoral cortex and the button by radiograph postoperatively.

### Statistical analyses

Summary statistics for each variable were created using frequencies and proportions for categorical data and means and standard deviations (SD) for continuous variables.

The change in graft tension (ΔGT) every 10 cycles was calculated as follows: ΔGT = 40 - graft tension after 10 times knee flexion and extension (N). The Shapiro-Wilk test revealed a non-normal, distribution of ΔGT, directing non- parametric statistics for analysis. We compared ΔGT in the AM and PL bundle every 10 cycles using the Friedman test and Wilcoxon signed-rank test with Bonferroni correction.

We summed ΔGT every 10 cycles as the total ΔGT. A linear regression model was used to investigate the relationship between total ΔGT in AM and PL bundle and age, sex, and graft diameter. Sex was treated as a binary variable and divided into females and males, and the regression coefficient of females relative to that of males was calculated. We selected AM bundle graft diameter as an exploratory variable to calculate the regression coefficient between ΔGT in the AM bundle and graft diameter. Similarly, we selected the PL bundle graft size as an exploratory variable to calculate the regression coefficient between ΔGT in the PL bundle and graft diameter. To ensure a power of 80% and a two-sided alpha level of 0.05, a minimum of 32 patients were required to reach statistical significance.

All *p*-values were two-tailed, and statistical significance was set at *p* < 0.05. Data management and analysis were performed using R statistical software (version 3.5.1).

## Results

Two patients received the gracilis tendon as a graft: one patient had a medial collateral ligament injury and the other had an avulsion fracture. Ultimately 39 patients were included in this study. We analyzed 24women and 15men with a mean age at the surgery of 27.4 years (range 14–51 years). Patient characteristics and surgical information are presented in Table 1.

**Table 1:** Patients characteristic and surgical information

Patients number	39
Age (y)	27.4 ± 12.4
Sex	
Male	14 (35.9)
Female	25 (64.1)
BMI (Kg/m^2^)	23.8 ± 3.6
Injured side	
Left	19 (48.7)
Right	20 (51.3)
Graft size (mm)	
AM bundle	5.8 ± 0.4
PL bundle	5.6 ± 0.3

Data were represented as a number (rate) and mean ± standard deviation

*BMI* Body mass index, *AM* anteromedial, *PL* posterolateral

**Fig. 2:**
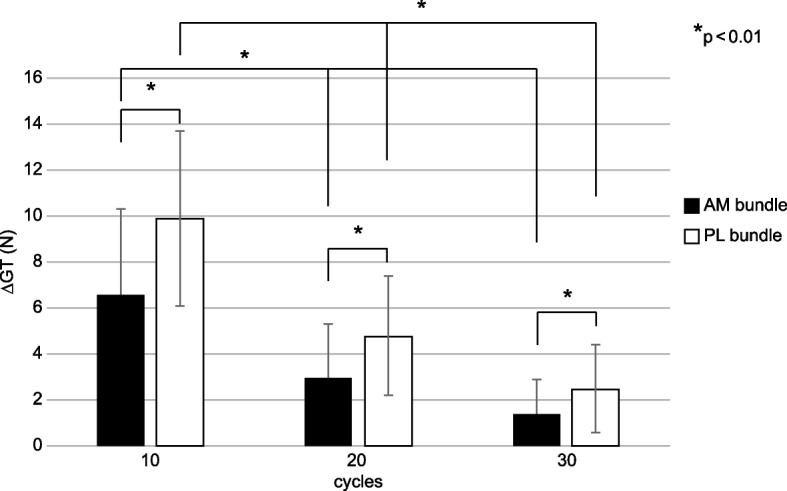
The change in graft tension every 10 cycles of knee flexion and extension

The mean ± SD and median ΔGT in the AM bundle at every 10 cycles were 6.6 ± 3.7 N and 6.0 N (interquartile range, 4.0 to 8.5), 3.0 ± 2.3 N and 2.0 N (interquartile range, 1.0 to 4.0), and 1.4 ± 1.5 N and 1.0 N (interquartile range, 0.0 to 2.0), respectively. The mean ± SD and median ΔGT in the PL bundle at every 10 cycles were 9.9 ± 3.8 N and 10.0 N (interquartile range, 6.5 to 12.0), 4.9 ± 2.6 N and 5.0 N (interquartile range, 3.5 to 5.5), and 2.5 ± 1.9 N and 2.0 N (interquartile range, 1.0 to 3.0), respectively. The mean ΔGT in the AM and PL bundles significantly decreased with repeated knee flexion and extension (*p* < 0.01). In addition, ΔGT in the AM bundle was significantly lower than that in the PM bundle during the same cycles (*p* < 0.01) (Fig. 2).

Table 2 presents the results of correlation analyses. No significant correlation was found between age, sex, graft size, and total ΔGT in the AM and PL bundles.

**Table 2:** Correlation analysis between total ΔGT and age, sex, and graft diameter

	AM bundle			PL bundle		
	β	SE	*p*-value	β	SE	*p*-value
Age	0.01	0.09	0.96	0.01	0.09	0.88
Sex [vs male]	1.25	2.58	0.63	−2.50	2.41	0.31
Graft diameter	3.87	3.04	0.21	5.49	2.94	0.07

*AM* anteromedial, *PL* posterolateral, *SE* standard error, *ΔGT* the change in graft tension

## Discussion

The most significant finding of our study was that cyclic knee motion intraoperatively reduced changes in soft tissue graft tension during primary double-bundle ACL reconstruction. Other important findings are that patient characteristics and surgical information are not associated with soft tissue graft tension change. Intra-operative cyclic knee motion is a convenient technique for deflecting the graft and creating graft tension appropriate for ACL reconstruction. A cyclic load may eliminate knot loosening between the thread and graft and elongate the graft. In addition, the viscoelastic creep of the graft was generated by repetitive tensile loading before graft fixation.

The current study showed that when the initial graft tension was 40 N, the AM, and PL graft tension decreased by 1.8 and 2.5 N after repeated knee flexion and extension at 30 cycles, respectively. Despite the importance of initial graft tension during ACL reconstruction, there is a paucity of evidence regarding the number of surgeons who should repeat knee flexion and extension. Several studies on graft tension have been conducted in biomechanical laboratories. One laboratory study reported that bone tendon-bone graft tension was stable after 500 cycles [11]. Intra-operative knee flexion and extension for up to several hundred cycles are difficult to achieve in clinical situations. Jiang et al. investigated the lengthening of hamstring grafts using cyclic knee motion in 53 patients who underwent single-bundle ACL and concluded that graft elongation achieved a stable level after 30 cycles [5]. Our results confirm that 3 sets of 10 cycle knee motion may suffice to obtain the initial graft tension that each surgeon aims to establish after ACL reconstruction.

Another finding was that the change in graft tension in the AM bundle was significantly smaller than that in the PL bundle in each repeated knee flexion and extension cycle. Several recent studies have shown different biomechanical reactions between the AM and PL bundles. In a normal ACL, the AM bundle is stretched in the full extension and flexion positions of more than 60°, whereas the PL bundle becomes slack in the flexion position [8]. A biomechanical study that assessed the graft tension patterns in the AM and PL bundles after ACL reconstruction revealed that the PL bundle was more tensioned than the AM bundle during knee flexion and extension between 0° and 30°^8^. Another in vivo study indicated that the PL bundle significantly contributed to the resistance to rotational forces compared with the AM bundle at 0° and 30° knee flexion [12]. The PL bundle may have been more susceptible to loosening than the AM bundle owing to cyclic knee motion because of the complex forces it was subjected to during knee flexion and extension between 0 °and 30 degrees.

The present study showed no significant association between graft tension changes caused by cyclic knee motion and patient characteristics during double-bundle ACL reconstruction. Furthermore, our findings are consistent with those of Jiang et al., who reported that there is no association between hamstring graft elongation and sex, age, or graft diameter during single-bundle ACL reconstruction [5]. In addition, they reported that graft elongation did not correlate with height, weight, affected side, duration of surgery, or graft length. Jiang et al. analyzed nine variables for 53 cases analyzed, which resulted in low statistical reliability. We performed our analysis using three variables for 39 cases and obtained results similar to those of a previous report. However, as graft fixation angles affect graft tension during knee motion [13, 14], we fixed the AM bundle at 30 degrees of knee flexion and the PL bundle at full knee extension. However, it was not possible to assess other fixation angles in clinical situations. Therefore, the association between graft tension changes caused by cyclic knee motion and graft fixation angles remains unknown.

This study has several limitations. The major limitation was that differences in surgical techniques could potentially influence changes in graft tension. This study was the limited surgical technique exhibited by using a hamstring tendon graft, an adjustable loop device, and an outside-in technique for ACL reconstruction. A previous laboratory study showed that cyclic knee motion caused the elongation of bone-tendon-bone grafts [11]. However, it is uncertain whether similar results can be obtained in clinical settings. The standard femoral fixation devices for soft tissue grafts are fixed-and adjustable loop device [15]. One biomechanical study reported that the adjustable-loop device lengthened under cyclic loads compared with a fixed-loop device [16]. Therefore, the generalizability of our results is subject to several limitations. In addition, we performed three sets of 10 cycles of knee flexion and extension to investigate changes in intraoperative graft tension; however, it is unclear whether a single set of 30 cycles of knee flexion and extension would yield similar results. Furthermore, we did not know if these graft tension changes are clinically relevant, as there was no measurement of postoperative knee laxity after ACL reconstruction. Finally, this study had a small sample size. Despite these limitations, the present study contributes to our understanding of cyclic knee motion during ACL reconstruction.

## Conclusions

Graft tensile loosening was observed after each intra-operative cyclic knee movement. The amount of loosening decreased after the retention and repeated cycles. Thus, this study suggests that repeated knee flexion and extension for 30 cycles may be sufficient to obtain a good initial hamstring graft tension during double-bundle ACL reconstruction.

## Data Availability

Requests for data not shown in the body of this manuscript can be made to the corresponding author.

## Abbreviations

ACL: Anterior cruciate ligament
ΔGT: The change in graft tension
AM: Anteromedial
PL: Posterolateral
SD: Standard deviation

## References

[1] Beasley LS, Weiland DE, Vidal AF, Chhabra A, Herzka AS, Feng MT (2005). Anterior cruciate ligament reconstruction: a literature review of the anatomy, biomechanics, surgical considerations, and clinical outcomes. Oper Tech Orthop.

[2] Mae T, Shino K, Nakata K, Toritsuka Y, Otsubo H, Fujie H (2008). Optimization of graft fixation at the time of anterior cruciate ligament reconstruction. Part I: effect of initial tension. Am J Sports Med.

[3] Koga H, Muneta T, Yagishita K, Watanabe T, Mochizuki T, Horie M (2015). Effect of initial graft tension on knee stability and graft tension pattern in double-bundle anterior cruciate ligament reconstruction. Arthroscopy..

[4] Howard ME, Cawley PW, Losse GM, Johnston RB (1996). Bone-patellar tendon-bone grafts for anterior cruciate ligament reconstruction: the effects of graft pretensioning. Arthroscopy..

[5] Jiang D, Ao YF, Jiao C, Guo QW, Xie X, Zhao F (2019). The effect of cyclic knee motion on the elongation of four-strand hamstring autograft in anterior cruciate ligament reconstruction: an in-situ pilot study. BMC Musculoskelet Disord.

[6] Sasho T, Sasaki T, Hoshi H, Akagi R, Enomoto T, Sato Y (2018). Evaluating different closed loop graft preparation technique for tibial suspensory fixation in ACL reconstruction using TightRope™. Asia Pac J Sports Med Arthrosc Rehabil Technol.

[7] Lee CH, Huang GS, Chao KH, Wu SS, Chen Q (2005). Differential pretensions of a flexor tendon graft for anterior cruciate ligament reconstruction: a biomechanical comparison in a porcine knee model. Arthroscopy..

[8] Yasuda K, Tanabe Y, Kondo E, Kitamura N, Tohyama H (2010). Anatomic double-bundle anterior cruciate ligament reconstruction. Arthroscopy..

[9] Siebold R, Cafaltzis K (2010). Differentiation between intraoperative and postoperative bone tunnel widening and communication in double-bundle anterior cruciate ligament reconstruction: a prospective study. Arthroscopy..

[10] Boyle MJ, Vovos TJ, Walker CG, Stabile KJ, Roth JM, Garrett WE (2015). Does adjustable-loop femoral cortical suspension loosen after anterior cruciate ligament reconstruction? A retrospective comparative study. Knee..

[11] Arnold MP, Lie DTT, Verdonschot N, De Graaf R, Amis AA, Van Kampen A (2005). The remains of anterior cruciate ligament graft tension after cyclic knee motion. Am J Sports Med.

[12] Zantop T, Herbort M, Raschke MJ, Fu FH, Petersen W (2007). The role of the anteromedial and posterolateral bundles of the anterior cruciate ligament in anterior tibial translation and internal rotation. Am J Sports Med.

[13] Koga H, Muneta T, Yagishita K, Ju YJ, Mochizuki T, Horie M (2013). Effect of posterolateral bundle graft fixation angles on graft tension curves and load sharing in double-bundle anterior cruciate ligament reconstruction using a transtibial drilling technique. Arthroscopy..

[14] Koga H, Muneta T, Yagishita K, Ju Y-J, Sekiya I (2012). The effect of graft fixation angles on anteroposterior and rotational knee laxity in double-bundle anterior cruciate ligament reconstruction: evaluation using computerized navigation. Am J Sports Med.

[15] Ranjan R, Gaba S, Goel L, Asif N, Kalra M, Kumar R (2018). In vivo comparison of a fixed loop (EndoButton CL) with an adjustable loop (TightRope RT) device for femoral fixation of the graft in ACL reconstruction: a prospective randomized study and a literature review. J Orthop Surg (Hong Kong).

[16] Barrow AE, Pilia M, Teja G, Kadrmas WR, Burns TC (2014). Femoral suspension devices for anterior cruciate ligament reconstruction: do adjustable loops lengthen?. Am J Sports Med.

